# Carcinogen 7,12-dimethylbenz[a]anthracene-induced mammary tumorigenesis is accelerated in Smad3 heterozygous mice compared to Smad3 wild type mice

**DOI:** 10.18632/oncotarget.11713

**Published:** 2016-08-30

**Authors:** Zhengxue Liu, Tanima Kundu-Roy, Isao Matsuura, Guannan Wang, Yong Lin, You-Rong Lou, Nicola J. Barnard, Xiao-Fan Wang, Mou-Tuan Huang, Nanjoo Suh, Fang Liu

**Affiliations:** ^1^ Center for Advanced Biotechnology and Medicine, Rutgers, The State University of New Jersey, Piscataway, NJ, USA; ^2^ Susan Lehman Cullman Laboratory for Cancer Research, Department of Chemical Biology, Ernest Mario School of Pharmacy, Rutgers, The State University of New Jersey, Piscataway, NJ, USA; ^3^ Rutgers Cancer Institute of New Jersey, New Brunswick, NJ, USA; ^4^ Department of Biostatistics, School of Public Health, Rutgers, The State University of New Jersey, Piscataway, NJ, USA; ^5^ Department of Pathology, Rutgers Robert Wood Johnson Medical School, Piscataway, NJ, USA; ^6^ Division of Molecular Genomics and Medicine, National Health Research Institutes, Zhunan, Miaoli County, Taiwan; ^7^ Department of Pharmacology and Cancer Biology, Duke University Medical Center, Durham, NC, USA; ^8^ College of Life Science & Engineering, Chongqing Three Gorges University, Chongqing, China

**Keywords:** Smad3, TGF-ß, DMBA, carcinogenesis, breast cancer

## Abstract

Previous studies based on cell culture and xenograft animal models suggest that Smad3 has tumor suppressor function for breast cancer during early stages of tumorigenesis. In this report, we show that DMBA (7,12-dimethylbenz[*a*]anthracene), a chemical carcinogen, induces mammary tumor formation at a significantly higher frequency in the Smad3 heterozygous mice than in the Smad3 wild type mice. This is the first genetic evidence showing that Smad3 inhibits mammary tumor formation in a mouse model. Our findings support the notion that Smad3 has important tumor suppressor function for breast cancer.

## INTRODUCTION

TGF-ß potently inhibits cell proliferation. It also induces apoptosis in several cell types, such as breast epithelial cells. Accordingly, TGF-ß plays a major role in the inhibition of tumor formation during early stages of tumorigenesis. For example, expression of TGF-ß1 transgene suppresses mammary tumor formation [1]. Transgenic mice overexpressing a dominant-negative mutant TGF-ß type II receptor show enhanced tumorigenesis in the mammary gland in response to the carcinogen 7,12-dimethylbenz-[*a*]-anthracene (DMBA) [2].

Smad3 plays a major role in mediating TGF-ß growth inhibitory and apoptotic effects [3, 4]. Smad3 is essential for TGF-ß-induced transcriptional repression of c-myc, Bcl2, Id-1, and hTERT and transcriptional activation of the CDK inhibitors p15 and p21 [3, 4]. TGF-ß-induced growth inhibition and apoptosis is essentially lost in Smad3^−/−^ primary mammary epithelial cells [5]. In addition, TGF-ß/Smad3 upregulates the expression of a set of genes including Ephrin-A1, which is shown to contribute to the TGF-ß/Smad3 tumor suppressive effects in ER-positive breast cancer [6].

Smad3 expression levels are low in breast cancer stem cells from human patients [7]. Inhibition of Smad3/2 enhances tumorigenicity of human MCF10CA1h (low grade, invasive) breast cancer cell line, which is derived from the MCF10A cells; whereas overexpression of Smad3 reduces tumorigenicity [8]. Furthermore, knockdown of Smad3 significantly increases tumorigenicity of the MCF10CA1h cells, and the tumors are more aggressive [6].

Smad3 has been shown or suggested to have tumor suppressor functions in pediatric T-cell acute lymphoblastic leukemia, gastric cancer, colon cancer, pancreatic cancer, and hepatocellular carcinoma [9–13], and Smad3 point mutations have been identified in colon and pancreatic cancers [14–16 and references therein]. Smad3 is rarely mutated in breast cancer. Smad3 point mutation rate in breast cancer is 0.4% (4 mutations out of 952 cases analyzed) (TCGA provision data). Modulation of Smad3 levels are much more frequent events than mutations in breast cancer.

We attempted to address the question whether reduction of Smad3 levels promotes *de novo* tumor formation. Smad3^−/−^ mice cannot be used for analysis of spontaneous mammary tumor formation, as they do not survive beyond at most 8 months due to certain defects [13, 17, 18]. The Smad3^+/−^ (heterozygous) mice do not form spontaneous mammary tumors (data not shown).

Previous xenograft experiments showing Smad3 tumor suppressor function for breast cancer used the MCF10CA1h cell line [6, 8], which contains an oncogenic H-Ras mutation. Although Ras mutation is not a frequent event for breast cancer, activation of ERK, which is downstream of Ras, is a frequent event in breast cancer. We therefore considered to first test in a mouse model that contains an oncogenic Ras mutation. DMBA is a prototypical polycyclic aromatic hydrocarbon that has been used to promote mammary tumor formation in mouse [19]. DMBA induces oncogenic H-Ras mutation [19]. Mutation rate of H-Ras in DMBA-induced mouse mammary tumors derived from hyperplastic outgrowth lines is estimated to be ~ 60-80% [20]. It should be noted while an area of active research, there is no causal link between the exposure to polycyclic aromatic hydrocarbons and human breast cancer. The use of DMBA-induced mammary tumorigenesis is primarily relevant from the standpoint of the role of Smad3 as a tumor suppressor. We show in this study that DMBA-induced mammary tumor formation is accelerated in the Smad3^+/−^ mice compared to the Smad3^+/+^ mice.

## RESULTS AND DISCUSSIONS

The Smad3^+/−^ mice are in a mixed C57BL/6/129 genetic background [17]. Smad3^+/−^ mice were mated with each other to produce Smad3^+/+^ and Smad3^+/−^ mice. Each of the Smad3^+/+^ and Smad3^+/−^ female mice in cohorts was given 1.0 mg dose of DMBA by oral gavage once a week for 5 weeks, beginning at 5-6 weeks of age. A mammary tumor occurred as early as 13 weeks after the first dose of DMBA in one Smad3 heterozygous mouse. Tumor formation was monitored for six months after beginning DMBA.

All palpable tumors were collected, and were analyzed histopathologically after hematoxylin and eosin (H & E) staining. As shown in Table I, at the end of the experiments, 8 out of 23 Smad3 wild type mice developed tumors, whereas 17 out of 26 Smad3 heterozygous mice developed tumors. It should be noted that some mice did not survive to the end of the experiments due to the adverse effects of DMBA. This occurs to both Smad3 (wild type) and Smad3 (heterozygous) mice. These mice died or were sacrificed early, usually within 1-2 months after the initial dose of DMBA. They had not developed mammary tumors at the time of death (data not shown). These mice are not included in our counting of animal numbers.

**Table I:** Number of mice with tumors

Group	Number of mice	Number of mice with mammary tumors	% of mice with mammary tumors	Number of mice with a skin squamous cell carcinoma	Total number of mice with tumors
Smad3 (WT)	23	8	34.8%	0	8
Smad3 (Het)	26	16	61.5%	1	17

For the Smad3 wild type mice, each of the 8 mice developed one mammary tumor, total 8 mammary tumors in the Smad3 wild type group (Table II). For the Smad3 heterozygous mice, total 18 tumors were developed from the 17 mice. Among the 18 tumors, 17 tumors are mammary tumors, and another tumor turned out to be a skin squamous cell carcinoma (Tables I and II). Among the 17 Smad3 heterozygous mice, one mouse developed two mammary tumors, one from left mammary gland #2 and one from left mammary gland #4; 15 mice each developed one mammary tumor; and one mouse developed a skin squamous cell carcinoma (Tables I and II).

**Table II:** Total number of mammary tumors

Group	Number of mice with mammary tumors	Number of mice with two mammary tumors	Number of mice with one mammary tumor	Total number of mammary tumors
Smad3 (WT)	8	0	8	8
Smad3 (Het)	16	1	15	17

**Figure 1:**
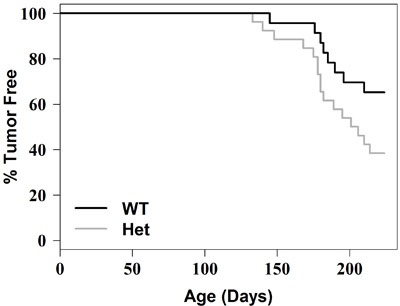
Tumor free survival curve Each of the 23 Smad3 wild type mice and 26 Smad3 heterozygous mice was given 1.0 mg dose of DMBA by oral gavage once a week for 5 weeks, beginning at 5-6 weeks of age. Tumor formation was monitored for six months (26 weeks) after beginning DMBA. Kaplan-Meier tumor free survival curve for Smad3 wild type mice (WT) and Smad3 heterozygous mice (Het) is shown. The p value is 0.0592 using log-rank test.

The Kaplan-Meier tumor free survival curve for Smad3 (WT) and Smad3 (heterozygous) mice is shown in Figure 1. The p value is 0.0592 using log-rank test. DMBA-induced mammary tumor formation is accelerated in the Smad3 heterozygous mice compared to the Smad3 wild type mice.

For mammary tumor incidence, the percentage of Smad3 wild type animals with mammary tumors is 34.8% (8 mice out of total 23 mice). The percentage of Smad3 heterozygous animals with mammary tumors is 61.5% (16 mice out of total 26 mice) (Table I). Mammary tumor incidence is significantly higher in the Smad3 heterozygous mice than in the Smad3 wild type mice (Chi-square test one sided p value is 0.03).

**Figure 2:**
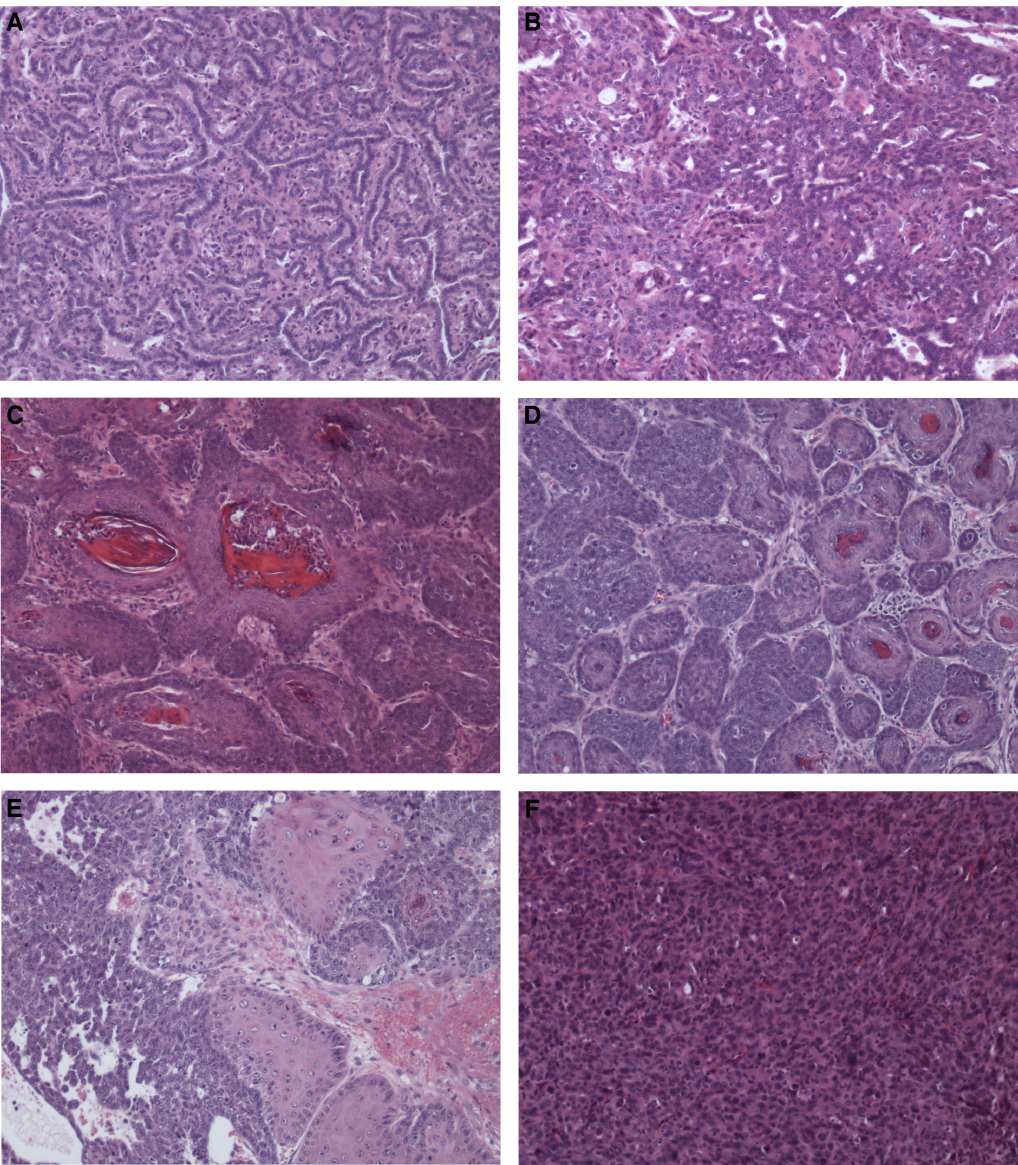
Examples of histology of mammary tumors **A.** A papillary adenocarcinoma from a Smad3 heterozygous mouse. This was the only Smad3 heterozygous mouse which developed a papillary adenocarcinoma. **B.** An adenocarcinoma from a Smad3 heterozygous mouse. **C.** A squamous cell carcinoma from a Smad3 heterozygous mouse. **D.** An adenosquamous carcinoma from a Smad3 wild type mouse. **E.** An adenosquamous carcinoma from a Smad3 heterozygous mouse. **F.** A poorly differentiated carcinoma from a Smad3 heterozygous mouse. Magnification 100X for all photos.

Histopathological analyses revealed that the mammary tumors were very heterogeneous. The mammary tumors were classified into papillary adenocarcinoma, adenocarcinoma, squamous cell carcinoma, adenosquamous carcinoma, and poorly differentiated carcinoma (Table III). Both the Smad3 wild type and heterozygous mice developed more adenosquamous carcinomas than other types of carcinomas (Table III). This is similar to previous studies showing that adenosquamous carcinomas occur more frequently than other types of carcinomas in DMBA-induced mammary tumors [for example, see references 2 and 21]. Examples of histology of the mammary tumors indicating papillary adenocarcinoma, adenocarcinoma, squamous cell carcinoma, adenosquamous carcinoma, or poorly differentiated carcinoma are shown in Figure 2.

**Table III:** Histopathological classification of mammary tumors

Group	Number of papillary adenocarcinomas	Number of adenocarcinomas	Number of squamous cell carcinomas	Number of adenosquamous carcinomas	Number of poorly differentiated carcinomas
Smad3 (WT)	0	1	1	5	1
Smad3 (Het)	1	3	4	8	1

**Figure 3:**
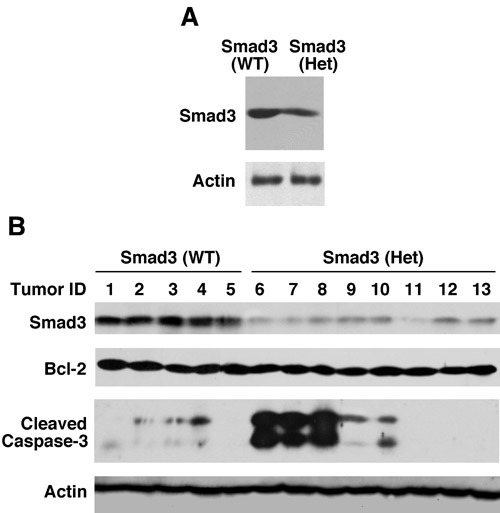
Cleaved caspase-3 is markedly increased in certain adenosquamous carcinomas from Smad3 heterozygous mice **A.** Smad3 protein levels are reduced in the normal mammary glands in the Smad3 heterozygous mice compared to the Smad3 wild type mice. A pool of three mammary glands from three 5-6 weeks old Smad3 wild type mice or Smad3 heterozygous mice were analyzed for Smad3 protein levels by Western blot. Actin expression levels were also analyzed as a control. **B.** Activated caspase-3 is markedly increased in three adenosquamous carcinomas from Smad3 heterozygous mice. Five Smad3 (WT) adenosquamous carcinomas and 8 Smad3 (Het) adenosquamous carcinomas were analyzed for the expression levels of Smad3, Bcl-2, and cleaved caspase-3. Actin expression levels were also analyzed as a control.

We confirmed that Smad3 protein levels were reduced in the normal mammary glands in the Smad3 heterozygous mice compared to the Smad3 wild type mice (Figure 3A). c-myc and Bcl-2 are important target genes of Smad3 based on cell culture studies. Their expression was not detectable in the normal mammary glands (data not shown).

Since the mammary tumors are in different types and we don't have sufficient number of tumors for each type (Table III), we focused on the analysis of the adenosquamous carcinomas, which are most abundant among all the types. The 5 adenosquamous carcinomas from the wild type group and the 8 adenosquamous carcinomas from the heterozygous group were analyzed for Smad3 levels. As shown in Figure 3B, the Smad3 protein levels were all reduced in the Smad3 (Het) tumors, compared to the Smad3 (WT) tumors. In particular, the Smad3 levels were very low in the Smad3 (Het) tumor #11 (Figure 3B). Interestingly, a previous study showed that Ras activation leads to downregulation of Smad3 at mRNA and protein levels in a breast cancer cell line [22]. It is possible that Smad3 (Het) tumor #11 contains an oncogenic Ras mutation, and this mechanism is applicable in Smad3 (Het) tumor #11.

A previous study showed that Smad3 repressed Bcl2 expression in immortalized mouse mammary epithelial cells (IMEC) [23]. Furthermore, it showed that the Bcl-2 levels were significantly higher in the Smad3 heterozygous IMEC than in the Smad3 wild type IMEC [23]. However, in the tumors that we analyzed, the Bcl-2 levels were similar between the wild type group and the heterozygous group (Figure 3B). We also analyzed c-myc expression levels. The c-myc levels were also similar between the wild type group and the heterozygous group, and the Ki67 staining was also similar between the two groups (data not shown).

Unexpectedly, when we analyzed the levels of cleaved caspase-3, we found that the activated caspase-3 levels were greatly increased in 3 of the 8 Smad3 (Het) adenosquamous carcinomas (Figure 3B). It should be noted that the tumors were not necrotic. The increased activated caspase-3 was not due to necrotic tumors. A recent study showed that mammalian cells, including breast epithelial cells, can survive with persistent caspase-3 activation, which causes limited DNA strand breaks [24]. This promotes genetic instability and oncogenic transformation [24, 25]. In addition, it has been shown that caspase-3 promotes skin carcinogenesis induced by DMBA + TPA treatment [24].

Although the activated caspase-3 increased greatly in the Smad3 (Het) tumors #6, #7, and #8, the apoptosis was not increased. In the TUNEL assay, the TUNEL positive cells were 1-3% in both the wild type group involving Smad3 (WT) tumors #1, #2, #3, and #4 and the heterozygous group involving Smad3 (Het) tumors #6, #7, #8, #9, and #10 (data not shown). This is consistent with the idea that persistent activation of caspase-3 can cause limited DNA breakage, leading to genetic instability and malignant transformation.

It should be noted that the Smad3 (Het) tumors #6, #7, and #8 do not show correlation with the tumor occurrence time, the size of the tumors, or the aggressiveness of the tumors. Analysis of more tumor samples in the future is necessary to establish whether there is a causal link. A related question is by which mechanism the reduced Smad3 levels lead to the markedly increased activated caspase-3 in those three Smad3 (Het) tumors.

Another strong message from the cleaved caspase-3 Western blot is that the tumors are extremely heterogenous. Three other Smad3 (Het) tumors contain very low levels of activated caspase-3 (Figure 3B). Approximately 70% of the DMBA-induced mammary tumors contain an oncogenic Ras mutation. The Ras pathway can inhibit Smad3 function through ERK and CDK phosphorylation of Smad3 [26–33]. Whether this activity in conjunction with reduced Smad3 levels has a role for the increased levels of activated caspase-3 remains to be determined. The remaining ~ 30% tumors contain other genetic and epigenetic changes. The diverse genetic and epigenetic background may account for in part the differences in the cleaved caspase-3 levels.

We also attempted to include Smad3^−/−^ mice in this study. Since a significant proportion of Smad3 null mice do not survive longer enough for tumor development after DMBA administration, we are not able to achieve statistically significant results (data not shown). In addition, since Smad3 null mice have certain defects [13, 17, 18], this complicates the interpretation of the data with the use of Smad3 null mice. Our results in this study suggest that Smad3 has important tumor suppressor function for breast cancer. In the future, it is warranted to examine whether Smad3 mammary null mice develop tumors and whether Smad3 mammary deficiency accelerates oncogene-driven or mutant p53-driven mammary tumorigenesis.

In conclusion, we have shown that deletion of only one copy of the Smad3 gene is sufficient to significantly increase the frequency of DMBA-induced mammary tumorigenesis. The tumors are extremely heterogeneous. Future studies are necessary to answer a number of important questions.

## MATERIALS AND METHODS

### Smad3^+/−^ and Smad3^+/+^ mice

The Smad3^+/−^ mice were previously described [17]. They are in a mixed C57BL/6/129 genetic background [17]. Smad3^+/−^ male and Smad3^+/−^ female mice were mated with each other. Genotyping of the mice was performed by PCR as previously described [17]. The resulting Smad3^+/+^ (wild type) and Smad3^+/−^ (heterozygous) female mice were used in this study.

### DMBA-induced mammary tumorigenesis

DMBA-induced mammary tumor formation was carried out essentially as previously described [21]. The Smad3^+/+^ and Smad3^+/−^ female mice in cohorts were each given 5 weekly 1.0 mg dose of DMBA in 0.1 ml of corn oil by oral gavage, beginning at 5-6 weeks of age. Tumor formation was monitored by palpation. Mice were monitored weekly for tumor formation in the first ten weeks, and twice per week in the subsequent weeks. Tumor formation was monitored for six months after the initial dose of DMBA. The animal protocols were approved by the institutional IACUC committee.

### Histopathology

Palpable tumors were dissected. They were fixed in 10% formalin/PBS and then preserved in 70% ethanol. Tumor samples were paraffin embedded, sectioned into slides with 4 micron thickness, and H & E stained by the Biospecimen Repository Service and Histopathology Shared Resources of the Rutgers Cancer Institute of New Jersey. All tumors were examined histopathologically after H & E staining. Mammary tumors were classified into papillary adenocarcinoma, adenocarcinoma, squamous cell carcinoma, adenosquamous carcinoma, and poorly differentiated carcinoma.

### Western blot analysis

Mammary glands or mammary tumors were homogenized in RIPA buffer, and the protein extracts were analyzed by Western blot analysis. The cleaved caspase-3 rabbit polyclonal antibody was from Cell Signaling Technology (Cat. No. 9661). The Bcl-2 rabbit monoclonal antibody was from Cell Signaling Technology (Cat. No. 2870). The Smad3 rabbit monoclonal antibody was from Abcam (Cat. No. 40854). The Actin mouse monoclonal antibody was from Sigma (Cat No. A1978).

### Statistical analysis

Statistical analysis was carried out using the log-rank test or Chi-square test.
